# Prevalence of complications associated with the use of a peripherally inserted central catheter in newborns: A systematic review protocol

**DOI:** 10.1371/journal.pone.0255090

**Published:** 2021-07-23

**Authors:** Edienne Rosângela Sarmento Diniz, Kleyton Santos de Medeiros, Richardson Augusto Rosendo da Silva, Ricardo Ney Cobucci, Angelo Giuseppe Roncalli

**Affiliations:** 1 Postgraduate Program in Public Health, Federal University of Rio Grande do Norte-UFRN, Natal, Brazil; 2 Health Sciences Postgraduate Program, Federal University of Rio Grande do Norte (UFRN), Natal, RN, Brazil; 3 Postgraduate Program in Sciences Applied to Women’s Health, Maternidade Escola Januário Cicco (MEJC / EBSERH), Federal University of Rio Grande do Norte, Natal, Brazil; University Magna Graecia of Catanzaro, ITALY

## Abstract

**Background:**

The improper handling of a peripherally inserted central catheter (PICC) in newborns (NBs) may result in mechanical and infectious complications.

**Aim:**

The aim of this systematic review (SR) is to estimate the prevalence of complications associated with the use of PICC in NBs.

**Methods:**

We will utilize PubMed, Embase, CENTRAL, Web of Science, Scopus, Cochrane Library, CINAHL, and Google Scholar for the databases search. There will be no restrictions on the search for languages, and observational studies will be selected wherein the prevalence rate of complications associated with the use of PICC in NBs has been presented or can be calculated. The systematic review will follow the guidelines of the Preferred Reporting Items for Systematic Reviews and Meta-Analyses. Two reviewers will independently select studies and assess their eligibility using predefined criteria. Using standardized forms, two other reviewers will independently extract data from each included study, and the random-effects pooled prevalence will be calculated in the meta-analysis with the respective 95% confidence intervals. The methodological quality of the studies will be assessed using the modified Newcastle-Ottawa Scale. Review Manager V.5.3.5 will be used for the qualitative and quantitative synthesis. A protocol was developed and published on PROSPERO (Registration number CRD42020211983).

**Expected results:**

This SR will show the prevalence of complications caused by the inadequate management of PICC in NBs, which is information considered important for clinical practice improvement.

## Introduction

The insertion of peripherally inserted central catheters (PICC) is common in neonatal intensive care units (NICU) in term newborns (NBs) and premature infants receiving venous therapies with vesicant and irritating drugs and parenteral nutrition. Moreover, depending on the caliber, it is also used for blood tests, transfusion of blood products, and hemodynamic monitoring [1, 2]. These reduce the risk of complications and dispense with the need for surgical intervention, which is advantageous due to the reduced hospitalization time, reduced hospital costs, and decreased incidence of neonatal mortality, as PICC reduce the incidence of complications when compared to other central catheters [3].

Premature NBs generally have low birth weight, and many are dependent on hyperosmotic or irritating drugs, which must be administered through a long-term venous access [2]. Previous studies have identified risk factors for complications associated with the use of PICC in NBs, including gestational age (GA), catheter insertion location, catheter tip location, and catheter dwell time, especially when it is installed for greater than 35 days, a factor also associated with an increased catheter-related bloodstream infection [1, 4]. Other mechanical complications appear with a lower incidence such as rupture of the catheter, migration of the catheter tip, obstruction, and leakage of drugs [5, 6]. These are responsible for several non-elective removals, and many of these can be avoided as they are related to the improper handling of the device [3, 7].

The use of PICC in NICU has become essential in neonatal clinical practice, and monitoring of the risk factors associated with complications is part of the neonatal care quality management in developed and developing countries, in which teams responsible for the quality management adopt evidence-based strategies to prevent these complications [8, 9]. Such complications can cause an increase in the costs of health services due to the increase in the length of stay in the NICU, as well as injuries to NBs, such as tissue, cardiological, and infectious injuries. Previous studies on complications related to the use of PICC in NBs have demonstrated the prevalence of tissue complications (edema, phlebitis, enlargement, and necrosis) and systemic complications (arrhythmias, cardiac tamponade, and sepsis) [3, 10–12].

Information on the complications associated with the use of PICC in NBs is scarce, and few studies that were conducted were observations done for short periods and focused on the risk factors associated with infection, but not on the prevalence of complications [8]. Thus, it is essential to develop studies to assess the prevalence of complications related to the use of PICC in NBs. The aim of this systematic review is to estimate the prevalence of complications associated with the use of PICC in NBs.

### Research question

What is the prevalence of complications associated with the use of PICC in NBs?

## Methods

This protocol was designed in accordance with the Preferred Reporting Items for Systematic Reviews and Meta-Analysis guidelines extension for reporting systematic review protocols (PRISMA-P) [13]. The protocol was registered with the International Prospective Register of Systematic Reviews (PROSPERO) (number: CRD42020211983). The systematic review will follow the guidelines of the Preferred Reporting Items for Systematic Reviews and Meta-Analyses (PRISMA) [14, 15].

### Ethics

Ethical approval is not required because this review will retrieve publicly available scientific literature. Traditional dissemination strategies will be used, including open-access peer-reviewed publications, scientific presentations, and reports.

### Inclusion criteria

This systematic review will include observational studies (case-control, cross-sectional, and cohort) that describe complications associated with the use of PICC in NBs. There will be no restrictions on the search for languages and the publication period.

### Exclusion criteria

Published articles, but not peer-reviewed articles, will not be included in the review. Randomized clinical trials, review articles, reports, and case series will be excluded. Studies that assessed the prevalence of complications in children (older than 28 days of life) will also be excluded.

### The PECOT strategy

- Population/participants: NBs
- Exposure: PICC
- Comparator/control: NBs who do not use the PICC
- Outcome: Complications associated with the use of PICC (mechanical, infectious, and systemic)
- Types of studies: observational studies (sectional, cohort, and case-control)

### Types of participants

Study participants will be NBs using PICC or not, neonates (children under 28 days of age), extremely premature (<28 weeks) NBs, very premature (28 to 31 weeks and 6 days) NBs, moderate or late preterm (32 to 36 weeks and 6 days) NBs, NBs with low birth weight, and healthy term NBs [16].

### Types of exposures

The included studies will be those describing complications in neonates using PICC, a peripherally inserted central catheter that is inserted through a peripheral vein and its tip is destined for the vena cava, used for medium-and long-term intravenous infusion or therapy with irritating and vesicant drugs [17, 18].

**Control**: NBs who do not use PICC.

### Outcomes

**Primary outcome:** Neonatal death
**Secondary outcomes:**
  - Pulmonary complications: pleural effusion, pneumothorax, and hydrothorax
  - Cardiovascular complications: arrhythmias, myocardial perforation, and cardiac tamponade
  - Tissue complications: hematomas, phlebitis, pain, local hardening, infiltration, leakage, and necrosis
  - Hematological complications: bleeding, embolism, and thrombosis
  - Infectious complications

### Types of studies

Observational studies: cross-sectional, cohort, and case-control.

### Search strategy

The studies will be obtained through PubMed, Embase, CINAHL, LILACS, CENTRAL, Web of Science, Scopus, Cochrane Library, and Google Scholar databases. There will be no restrictions on the search for languages and year of publication. Articles will also be searched from the references of the selected studies, and the search strategy used in PubMed is shown in Table 1.

**Table 1:** Search strategy for PubMed.

	MESH Terms
**1**	Infant, Newborn
**2**	Neonate
**3**	Newborn Infants
**4**	Newborn disease
**5**	Preterm Infant
**6**	Extremely Premature Infant
**7**	OR / 1–6
**8**	peripherally inserted central venous catheter
**9**	PICC Placement
**10**	Peripherally Inserted Central Catheter Line Insertion
**11**	Venous Catheterizations, Peripheral
**12**	OR / 8–11
**13**	Complications
**14**	catheter infection
**15**	Catheter-Related Infections
**16**	Embolism
**17**	Thrombosis
**18**	Bleeding
**19**	Arrhythmia
**20**	Necrosis
**21**	OR / 13–20
**22**	Observational Study
**23**	Cohort Studies
**24**	Case-control Studies
**25**	OR / 22–24
**26**	7 AND 12 AND 21 AND 25

### Data collection and analysis

#### Study selection

After searching the databases, all identified articles will be exported to Rayyan software and duplicates will be removed. First, the titles and abstracts will be read independently by at least two reviewers (ERSD and KSM) based on the inclusion criteria. The full texts of these potentially eligible studies will be retrieved and taken independently for eligibility by two members of the review team (ERSD and RNC). Only studies identified by both pairs of reviewers based on the inclusion criteria will ultimately be included in the systematic review, and a third reviewer (RAS) will make a final decision for inclusion in case of discrepancy.

**Fig 1:**
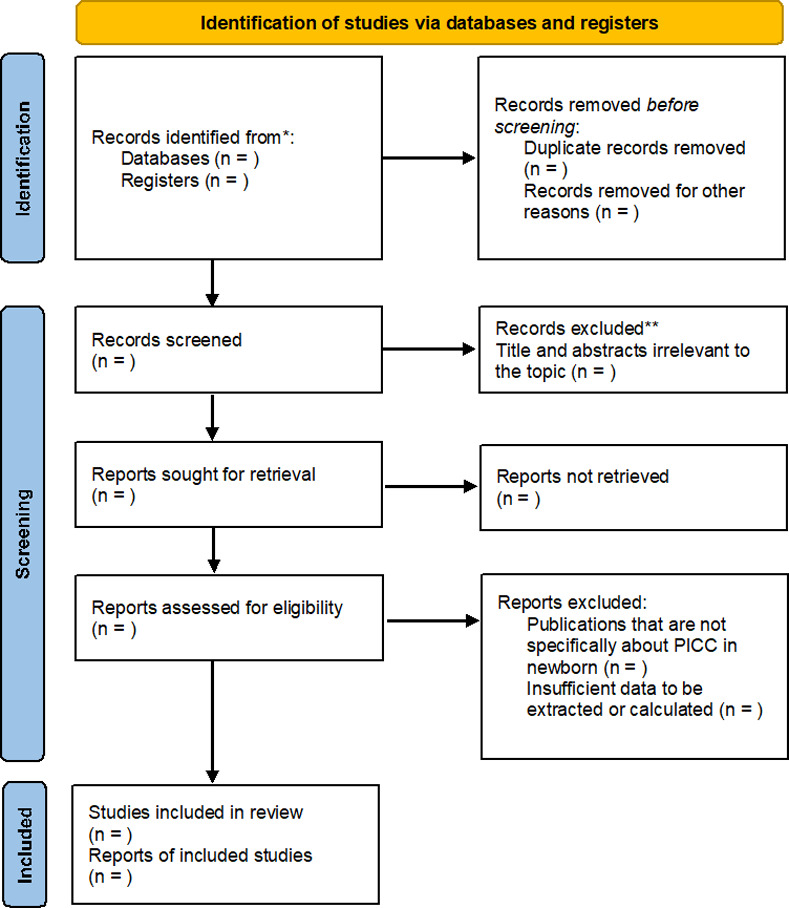
PRISMA flow diagram for systematic review and meta-analysis.

We will maintain a record of the reasons for excluding clinical trials at all stages of the review. The results of the selection or exclusion of the studies will be reported using the PRISMA flowchart, as shown in Fig 1.

#### Data extraction

Using standardized forms, two reviewers (ERSD and KSM) independently will extract the following data from each included study: first author, year, place of study, population and sample, type of study, objective, design, variables analyzed (GA of the NB, NB weight, NB diagnosis, catheter insertion location, use of parenteral nutrition, use of vasoactive drugs and antibiotics, time of use of PICC), and complications associated with the use of PICC. The extracted data cover the issue of the review and will be verified again by three authors (RNC, RAS, and AGR).

#### Addressing missing data

In case of a lack of data, the authors of this article will contact the respective authors or co-authors of the article in question by telephone or e-mail. If information will not be received, the data will be excluded from our analysis and will be covered in the discussion section.

#### Quality assessment of the included studies

The methodological quality of the selected studies will be assessed using the modified Newcastle-Ottawa Scale [19]. The following items will be analyzed: exposure, comparability, sample representativeness, sample size, response rate, outcome assessment, and statistics. The classification of the methodological quality of the studies will be carried out considering the total number of points received: ≥ 4 for good quality and <4 for low quality. The divergences found will be discussed and resolved by 3 authors of the review (ERSD, RNC, and LNS) [20].

### Assessment of heterogeneity

#### Measures of treatment effect

The results of the systematic review will be written in a structured manner with respect to the characteristics of the target population, type of primary outcomes (neonatal death), and secondary outcomes (systemic and tissue complications, rupture of the catheter and others).

The general prevalence of complications in NBs with PICC will be calculated using the random effects model, considering the heterogeneity between the studies included in the review. Cochran’s Q test will be used to assess heterogeneity and the I² statistic for quantification. The result of I² ≤ 50% will be considered as low heterogeneity and, in this case, the fixed effects model will be used. For I²> 50%, high heterogeneity, the random effects model will be used to calculate the prevalence ratio and 95% confidence interval (95% CI). When possible, we will use Egger’s funnel plot to assess possible publication bias.

#### Data synthesis

A quantitative synthesis (meta-analysis) will be performed in the RevMan 5.3.5 software using the inverse variance method with the random effects model if there is more than 50% heterogeneity between the studies. In cases where the data will be insufficient to calculate an effect estimate, a narrative synthesis will be created, describing the direction and size of the effects, along with any reported accuracy measures.

#### Subgroup and sensitivity analysis

Sensitivity analysis will be conducted to verify possible sources of heterogeneity, removing one study at a time, and verifying if there is a considerable change in the prevalence estimate and 95% CI. Sensitivity analysis will be performed excluding studies with a high risk of bias. When the effect estimates of the primary and sensitivity analyses are significantly different, an adjusted sensitivity analysis will be performed.

We plan to perform the following subgroup analyses, wherever possible: type of complication found (local versus systemic), GA, weight, diagnosis, days of life, and indications for the use of PICC. If we identify significant differences between the subgroups (test for interaction <0.05), we will report the results for individual subgroups separately. We will also perform a formal test for subgroup interactions using the RevMan version 5.3.5.

### Grading quality of evidence

#### Assessment of certainty of evidence

The analysis of the evidence for all outcomes will be assessed using the Grading of Recommendations Assessment, Development and Evaluation Working Group (GRADE) methodology [21] by classifying the evidence as high, moderate, low, or very low.

## Discussion

Knowledge about the actual prevalence of complications resulting from the use of PICC is essential for clinical practice in NICU and may allow the adoption of strategies that reduce those that are more serious, which can lead to the death of NBs. Previous studies have revealed a strong association between the time of NBs exposure to PICC during administration of parenteral nutrition and the use of antimicrobials with the development of complications, especially bloodstream infection [22–24]. However, systematic reviews of the prevalence of these complications are scarce.

A previous meta-analysis revealed that PICC inserted in the lower limbs did not show worse results compared to the upper limb group in the NICU, apart from thrombosis [25]. A finding divergent from this was demonstrated by Pet et al., who reported the occurrence of complications more frequently with PICC inserted in the upper extremities than in the lower extremities [26]. In another study, it was observed that the insertion of the PICC in the first 48 hours of life did not increase the prevalence of complications [12].

A recent study conducted in Greece revealed that the complication rates were similar when comparing the types of catheters used in NBs with low birth weight, and the authors recommend that central venous catheters should be removed early in NICU [27]. Studies have shown that low birth weight is a risk factor for complications associated with PICC in the NICU, and Wen et al. demonstrated that infectious and non-infectious complications of PICC are associated with low weight gain in premature babies [27, 28]. However, there is no reliable data on the actual prevalence of complications of PICC use in premature or low birth weight infants.

Biofilm is a fundamental component in the pathogenesis of infectious complications of PICC. Biofilm can be the cause of PICC extraction and can lead to serious haematogenic infectious complications that can increase the morbidity and mortality of affected babies. In order to help physicians and nurses to better target their preventive and therapeutic measures, will be important to understand which organism has the greatest impact on the development of PICC related bloodstream infections and to study the prevalence of the conditions worldwide, since effective prevention represent a sensitive target to reduce the prevalence of infectious complications during the use of PICC [29].

The potential limitations of the systematic review will focus on several aspects of the study design, searches, and quality appraisal of included studies. Limitations are related to include cross-sectional and case-control studies to hinder the reliable assessment of the causal relationship between PICC and complications in NBs. Furthermore, a small sample size and a limited number of studies can influence the validity and reliability of the findings.

Therefore, this systematic review will be carried out using a specific approach with a meta-analysis of the included studies results if possible. It is justified because knowing the prevalence of complications associated with the use of PICC in NBs in the NICU can positively impact the practice of care for the NB during infusion therapy and allow the creation of strategies to reduce serious complications such as sepsis and death. We expect that it will show the prevalence of complications caused by the inadequate management of PICC in NBs, which is information considered important for clinical practice improvement.

## Supporting information

S1 Checklist Preferred Reporting Items for Systematic review and Meta-Analysis Protocols (PRISMA-P checklist). (DOCX)
